# Effectiveness of using platelet‐rich fibrin to increase keratinized tissue around the tooth in the modified apical reposition flap method: A split‐mouth, randomized controlled trial

**DOI:** 10.1002/cre2.693

**Published:** 2022-11-24

**Authors:** Parviz Torkzaban, Nazli Rabienejad, Zahra Cheraghi, Kamran Lahoorpoor

**Affiliations:** ^1^ Department of Periodontology, Faculty of Dentistry Hamadan University of Medical Sciences Hamadan Iran; ^2^ Department of Epidemiology Hamadan University of Medical Science Hamadan Iran

**Keywords:** attached gingiva, MARF, PRF

## Abstract

**Objectives:**

One of the simplest methods to increase keratinized gingiva is the modified apically repositioned flap (MARF) technique. In this method, the periosteum remains exposed, which may lead to postoperative pain and discomfort. In the presence of bone dehiscence, bone resorption and gingival recession may occur. Hence, this study aims to use platelet‐rich fibrin (PRF) to promote wound healing in the MARF technique and overcome its disadvantages.

**Material and Methods:**

In this randomized controlled trial study, 10 patients (six males and four females with a mean age of 33.9 ± 11.13) with less than 2 mm of attached gingiva bilaterally were treated by the MARF + PRF membrane (test group), on the one hand, whereas, on the other hand, it was treated only by MARF (control group). Clinical parameters of keratinized gingiva include thickness, width, and vestibule depth before and 8 weeks after the intervention were measured.

**Results:**

The attached gingival width increased significantly in both groups (1.7 mm in the MARF and 2.3 mm in the PRF) and this was greater in the PRF group (*p* < .05). Gingival thickness in the PRF method was significantly higher than in the control group (*p* < .05). Probe depth, vestibular depth, and postoperative pain were not different in both groups. Wound shrinkage in the MARF group (51%) was significantly higher than in the PRF group (30%) (*p* < .05).

**Conclusion:**

Using PRF with the MARF method significantly increased the width and thickness of the gingiva and reduced shrinkage compared to MARF only. Postoperative pain and vestibular depth changes were similar in both groups.

## 1 INTRODUCTION

Gingiva is the part of oral mucosa that covers the alveolar processes of jaws and surrounds the neck of the teeth. The gingiva is divided anatomically into marginal (unattached), attached, and interdental areas (Newman et al., 2020). The attached gingiva contains a keratinized epithelium and, along with the dense bundles of collagen fibers that are firmly inserted into the cementum and/or into the underlying bone, serves as an effective barrier. The attached gingiva is very important for maintaining the health and hygiene of teeth and implants. The keratinized gingiva is the part of the oral mucosa that covers the gingiva and hard palate. It extends from the free gingival margin to the mucogingival junction and consists of the free gingiva as well as the attached gingiva. The width of the keratinized gingiva includes marginal and attached gingiva and differs in different areas of the mouth (Newman et al., 2020). Various authors consider at least 2 mm of keratinized gingiva around the teeth to be necessary to maintain periodontal health (Lang & Löe, 1972; Miyasato et al., 1977; Wennström et al., 1982). Gingival biotype was associated with age, gender, and keratinized gingival width (Kiani et al., 2021). Numerous studies have shown the negative effect of the absence of attached gingiva on plaque accumulation (Adibrad et al., 2009; Chung et al., 2006; Zigdon & Machtei, 2008). Other studies reported plaque accumulation, increased pocket depth, increased bleeding on probing, gingival resorption, and even marginal bone loss (Bouri et al., 2008; Boynueğri et al., 2013; Schrott et al., 2009). Alveolar bone loss, muscle and frenum attachments, and shallow vestibule are the causes of keratinized tissue deficiency (Stimmelmayr et al., 2011). Several methods have been introduced to increase the width of the attached gingiva around teeth and implants. One of the basic methods to increase the width of keratinized tissue is the apically repositioned flap, which was introduced by Friedman (1962). In this method, the mucoperiosteal flap with the adherent gingival tissue is moved to its apical position by two vertical incisions and fixed by sutures. The bone remains denuded and the wound would heal by means of secondary‐intention healing (Friedman, 1962). Due to the denudation of the coronal bone in the range of 3–5 mm, the risk of bone resorption was high. The main limitation of this technique was its side effects on the patient, including pain, bleeding, and bone resorption (Matter, 1982; Staffileno, 1969). Alternatives have been suggested. Free gingival grafting (FGG), double papillary flap, and modified apically repositioned flap (MARF) are among these (Staffileno, 1969). MARF was introduced by Carnio in 1999 to increase attached gingiva for a single tooth (Carnio & Miller, 1999) and in 2006 for several teeth (Carnio & Camargo, 2006). In this technique, the periosteum remains exposed instead of the bone. The advantages of this method are simplicity, minimal patient discomfort, and high color matching (Carnio & Camargo, 2006).

In this method, a horizontal incision is performed in the attached gingiva and a split‐thickness flap is raised and sutured apically. The periosteum remains exposed, the granulation tissue forms on top of the wound, and eventually, the keratinized tissue will mature. Leaving the periosteum naked may lead to postoperative pain and discomfort. In the presence of bone dehiscence, marginal bone resorption and gingival recession may occur, which is a contraindication of this technique.

On the other hand, the patient's platelet derivatives have been used for many years to accelerate wound healing. Various generations have been introduced to date, the most widely used of which is the platelet‐rich fibrin (PRF) membrane (Choukroun et al., 2006a). Due to the disadvantages of the apical repositioning flap technique, to reduce or overcome the disadvantages of this technique, we utilized the PRF membrane as a biological dressing. The rationale for using PRF in this method is to release growth factors and accelerate wound healing so that the underlying bone will not be remodeled. PRF acts as a biological dressing. Therefore, this study aims to use the PRF membrane as an adjunct to the MARF to reduce pain, swelling, resorption, and also to increase the width of the attached gingiva compared to the MARF technique.

## 2 MATERIALS AND METHODS

### 2.1 Study design

This study was designed as a randomized controlled trial with a split‐mouth design. To participate in the study, the following inclusion and exclusion criteria need to be fulfilled. This study was done to evaluate the clinical results of using PRF in combination with MARF to increase the attached gingiva around the teeth.

#### 2.1.1 Inclusion criteria

Inclusion criteria in the study were: patients with less than 2 mm of attached gingiva at the buccal site; less than 5 mm of vestibular depth (VD); and the presence of high frenum pull bilaterally in the mandible.

#### 2.1.2 Exclusion criteria

Exclusion criteria were systemic diseases, pregnancy and lactation, smoking, alcohol use, plaque index >20%, gingival recession Class 3 or 4 Miller, and PPD >5 mm.

Patients were invited to participate in the study after being fully informed. Informed consent was obtained from all participants. The study was approved by the Ethical Committee of the Hamadan University of Medical Science. The approval ID is IR.UMSHA.REC.1400.114.

### 2.2 Participants and randomization

The present study was performed in the periodontics department of the Dentistry School of Hamadan University of Medical Sciences from May to September 2021; 10 people who had less than 2 mm of attached gingiva and needed soft tissue augmentation on both sides were selected.

The sample size was determined based on the assumption that the test group (MARF + PRF) and the control group (MARF) were equal. Mean and standard deviation indices were extracted from a similar study by Temmerman et al. (2018) The confidence level was set at 95%, the desired sample power was 80%, using Stata (StataCorp. 2015. *Stata Statistical Software: Release 14*. College Station, TX: StataCorp LP.), and the sample size was determined to be 10 patients.

Each lower jaw was divided into two segments. Each side was randomized as control or test groups in a split‐mouth design, via a randomization table by a computer‐generated randomization list (STATA version 11). The treatment codes (test/control) were available in closed envelopes, which were sealed and opened just before the surgery by the surgeon. Blindness was not possible due to the nature of the procedure, and both the patient and the surgeon were informed about the treatment modality.

### 2.3 Surgical procedures

Chlorhexidine 0.2% was used for rinsing the oral cavity before surgery. Local anesthesia (lidocaine 2% with epinephrine 1:80,000) was performed in the buccal area of the premolars by mental nerve block.

#### 2.3.1 Control group (MARF)

**Figure 1:**
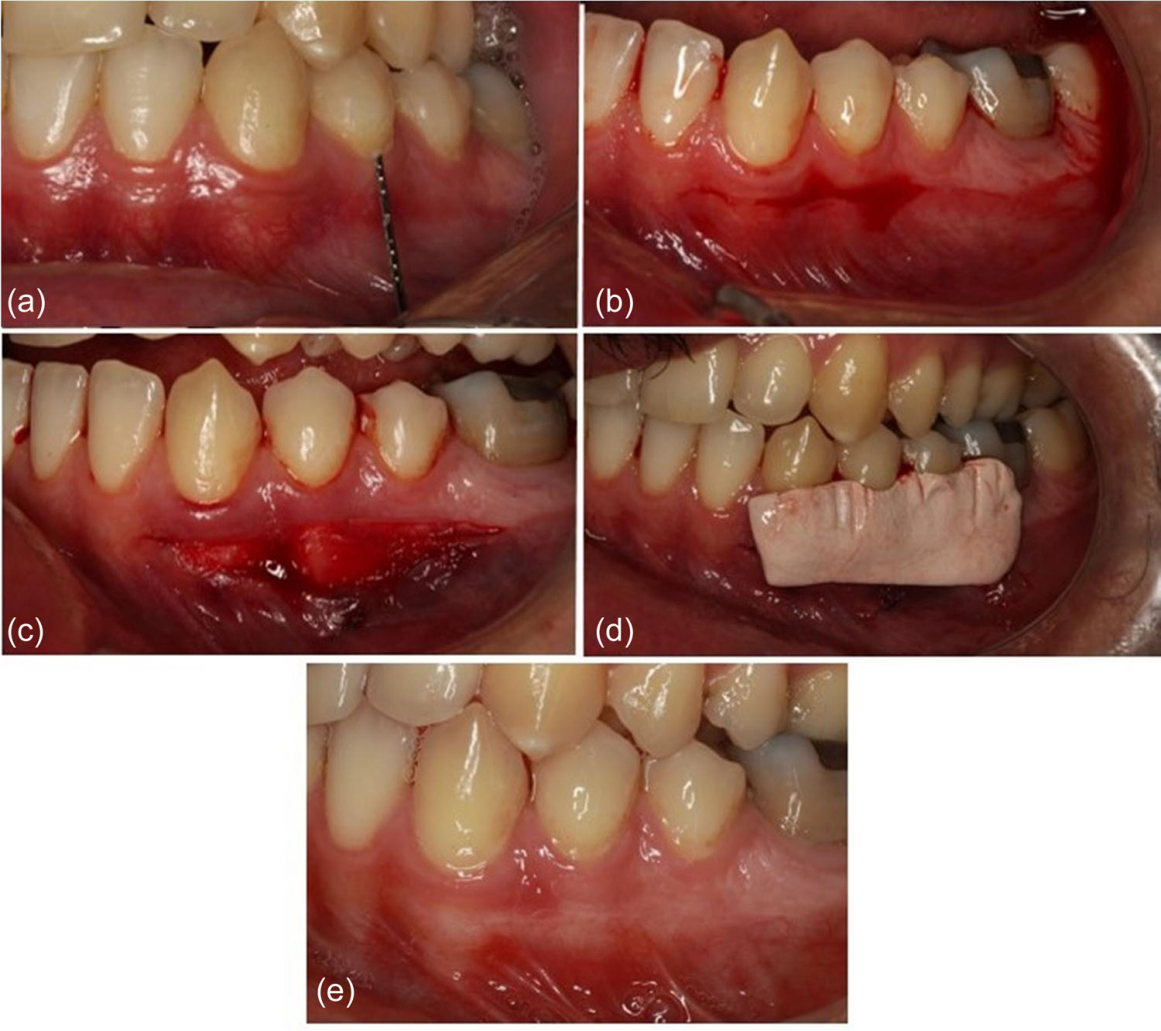
(a) Preoperative view. The first premolars with an attached gingiva width less than 2 mm. (b) A horizontal incision half a millimeter above the mucogingival junction line, with one‐and‐a‐half tooth extension. (c) Absorbable suture is used to secure the flap in the apical position. (d) Placement of periodontal dressing to protect the wound. (e) Eight weeks postop.

A horizontal incision was made by a blade (no. 15) approximately 0.5 mm coronal to the mucogingival junction. The split‐thickness flap was elevated and the dissection was extended 5 mm in an apical direction. The coronal part of the flap would contain a band of keratinized tissue. The extension of the incision depends on the teeth involved so that at least one and a half teeth are extended on each side (mesiodistal). This extension allows the flap to be repositioned apically. The flap is sutured to the periosteum in the apical position by resorbable sutures (Vycril® 4.0; ETHICON, Cincinnati, Ohio, USA). A homogeneous layer of periosteum with no elastic or fibers remained on the bone. A periodontal dressing without eugenol (COE‐PAK™; GC Corporation, Alsip, Illinois, USA) was used on top to protect the wound (Figure 1).

#### 2.3.2 Test group (PRF)

Patient's blood was collected into 10 ml sterile silica‐coated plastic tubes without anticoagulants (VACUETTE; Greiner Bio‐OneOne GmbH, Kremsmünster, Austria). At least two tubes were prepared, and the tubes were centrifuged immediately in a fixed‐angle centrifuge (Arman Teb Noor, Iran, Tabriz). According to studies by R. Miron et al. (2018) and R. J. Miron et al. (2019a) to obtain a relative gravity force of 400 g relative centrifugal force (RCF) clot and device specifications (rotor angulation: 30° and radius at the clot: 60 mm), the centrifuge speed was considered to be 2400 rpm. PRF clots and membranes were prepared in the same manner as described by Choukroun et al. (2006a). The PRF clots are placed in their special container to be compressed and in the form of a membrane.

**Figure 2:**
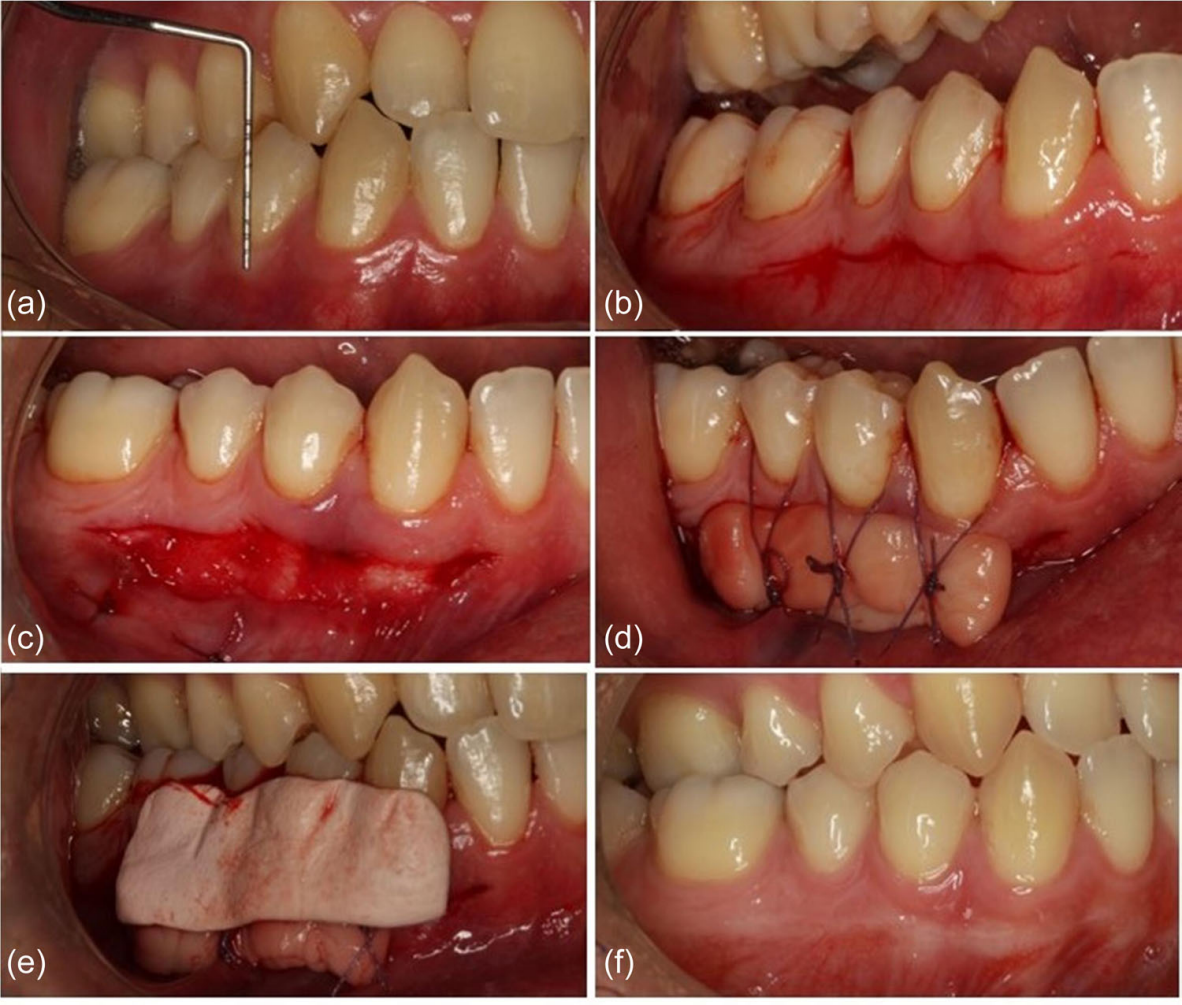
(a) Preoperative view. The first premolars with an attached gingiva width of less than 2 mm. (b) A horizontal incision half a millimeter above the mucogingival junction line, with one‐and‐a‐half tooth extension. (c) Absorbable suture is used to secure the flap in the apical position. (d) Placing and securing three layers of platelet‐rich fibrin membrane to the periosteum. (e) Periodontal dressing to protect the wound. (f) Eight weeks postop.

The MARF was done exactly as mentioned before. Several layers of PRF membranes (two or three layers) were placed on the exposed periosteum and were fixed by sutures around the teeth and finally dressed with a periodontal dressing (COE‐PAK™; GC Corporation, Alsip, Illinois, USA) (Figure 2).

All patients were prescribed 0.2% chlorhexidine mouthwash twice a day for 4 weeks, and ibuprofen 400 mg four times a day or as needed. The periodontal dressing and sutures were removed after 1 week. The patient was prohibited from doing any mechanical cleaning in the surgical area such as brushing and flossing for 4 weeks (Carnio et al., 2020).

### 2.4 Primary outcomes: Width and thickness of keratinized tissue

**Figure 3:**
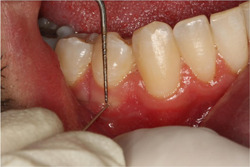
Preoperative measurement of gingival thickness

The patient's oral cavity was examined by a specialist with Williams Probe (Hu‐Friedy; Chicago, USA) to evaluate the clinical parameters. The evaluated parameters such as probing pocket depth (PPD), keratinized tissue width, VD, and gingival thickness (GT) were measured at the baseline, and after 8 weeks. GT measurements were taken using an endodontic spreader (#30) from 2 mm of the buccal gingival margin as shown in Figure 3 under local anesthesia (Kolte et al., 2014).

### 2.5 Secondary outcomes: Postoperative pain and wound shrinkage

A secondary objective of the study was to assess postoperative pain. A visual analog score (VAS) questionnaire was used to evaluate the amount of pain, ranging from 0 (no pain) to 10 (worst pain), and the patients were asked to fill out the questionnaire on the VAS scales on the day after surgery.

To assess the wound shrinkage, the amount of apical placement of the flap at the surgery was compared to the newly attached gingiva gained after 8 weeks. Wound shrinkage was calculated as follows:

(1) $$\frac{\text{apically displacement(in mm)}-\text{new attached gingiva gained}(\mathrm{in}\;\mathrm{mm})}{\text{apically displacement}}\times 100.$$

### 2.6 Statistical analysis

The data were checked for normality in distribution using a normal quantile plot. The confidence level was determined at 95%. The patient was used as a statistical unit. After a rejection of normality, a paired *t*‐test was used. The level of significance was set at .05.

## 3 RESULT

**Figure 4:**
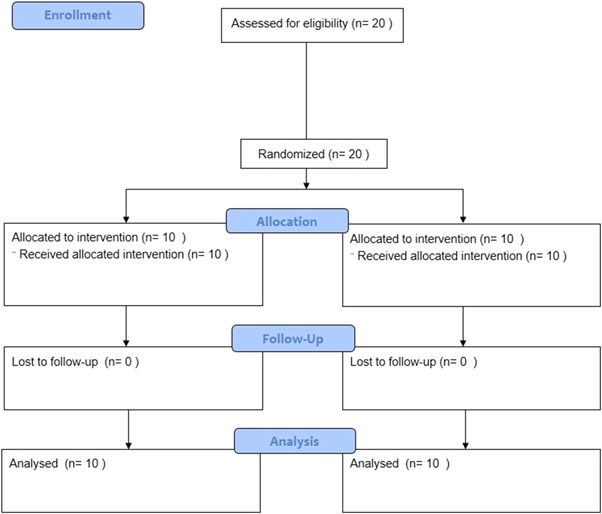
Flow diagram of the study

Ten patients participated in this trial. The mean age was 33.9 ± 11.1 years old and the male/female ratio was 6:4. None of the subjects dropped out during the 8‐week follow‐up. One side of each patient was randomly assigned to the test (MARF + PRF) or control (MARF) group, while the contralateral side was assigned to the other group. A flow diagram of the study participants is provided (Figure 4).

PPD at baseline and follow‐up were almost the same and there was no difference in either group.

At baseline, the average width of the attached gingiva was 1.15 ± 0.52 for the test group and 0.8 ± 0.82 for the control group. The mean increase in the attached gingiva after 8 weeks was 1.7 mm in the MARF group and 2.3 mm in the PRF group, which was significantly higher in the PRF group compared with the MARF group (*p* < .05).

At baseline GT was 0.79 ± 0.16 and 0.74 ± 0.22 mm for the test and control groups, respectively. After 8 weeks, the GT was 1.09 ± 0.09 in the PRF group and 0.86 ± 0.16 mm in the MARF group. GT is increased by 0.11 mm in the MARF group and 0.29 mm in the PRF group, with a significant statistical difference between those values (*p* < .05).

The VD at baseline and follow‐up are recorded in Table 1. Both treatment modalities lead to an increase in VD. The VD in the PRF and MARF groups increased by 1.9 and 1.4 mm, respectively. Although their differences were not significant (*p* > .05) (Table 1).

**Table 1:** Clinical measurements at baseline and 8‐week postop (mean ± SD) and clinical parameter change (Δ) from baseline to 8 weeks postop

Index	Mean ± SD		Group differences	*p* Value
	PRF group	MARF group	(mean ± SD)	
PPD				
Baseline	1.9 ± 0.5	1.80.8		
Follow‐up	1.9 ± 0.4	1.7 ± 0.9		
Δ	0 ± 0.5	0.1 ± 0.9	0.1 ± 0.4	.849
*p* Value	.811	.778		
AG				
Baseline	1.15 ± 0.52	0.8 ± 0.82		
Follow‐up	3.45 ± 0.49	2.5 ± 0.94		
Δ	2.3 ± 0.53	1.7 ± 0.97	0.6 ± 0.32	.049
*p* Value	.000	.002		
GT				
Baseline	0.79 ± 0.16	0.74 ± 0.22		
Follow‐up	1.09 ± 0.09	0.86 ± 0.16		
Δ	0.29 ± 0.15	0.11 ± 0.17	0.18 ± 0.07	.012
*p* Value	.000	.034		
VD				
Baseline	4 ± 0.52	4 ± 1.49		
Follow‐up	5.9 ± 0.45	5.4 ± 1.41		
Δ	1.9 ± 0.65	1.4 ± 1.04	0.5 ± 0.32	.143
*p* Value	.000	.001		

*Note*: Data were assessed with two‐sample *t*‐tests.

Abbreviations: AG, attached gingiva; GT, gingival thickness; MARF, modified apically repositioned flap; PPD, probing pocket depth; PRF, platelet‐rich fibrin; VD, vestibular depth.

Postoperative pain is assessed by using the VAS questionnaire a day after surgery. The VAS values associated with PRF and MARF groups were almost the same and were not different in either group (*p* > .05).

Wound shrinkage was measured by comparing the apicocoronal dimension of the exposed periosteum with the newly attached gingiva 8 weeks postop. The wound shrinkage was 51.1% in the control group and 30.9% in the test group, which was significantly higher in the MARF group (Table 2).

**Table 2:** Comparison postoperative pain and shrinkage

Index	Mean ± SD		*p* Value
	PRF group	MARF group	
Postoperative pain	3.20 ± 0.91	3.40 ± 0.69	.295
Wound shrinkage	30.90 ± 7.9	51.10 ± 20.30	.003

Abbreviations: MARF, modified apically repositioned flap; PRF, platelet‐rich fibrin.

## 4 DISCUSSION

A gingival width of 2 mm is sufficient to maintain healthy gingiva (Wennstrom & Lindhe, 1983). Lack of attached gingiva tissue along with inadequate plaque control may lead to a gingival recession (Freedman et al., 1992).

The apical repositioning flap introduced by Friedman increases the risk of bone resorption (Lang & Löe, 1972) and a regional accelerated phenomenon (Freedman et al., 1992). To overcome the problems of the apical repositioning flap, a modified method was proposed by Carnio and Miller in 1999. The MARF has more advantages over routine techniques for augmenting attached gingiva. This technique does not lead to further attachment loss, has predictable results, causes minimal discomfort to the patient due to the lack of the need for a donor site, and prevents gingival recession compared to the classic apical repositioning flap (Carnio & Miller, 1999; Carnio et al., 2007).

In a 1‐ to 11‐year follow‐up study, Carnio et al. (2020) evaluated the potential of creating attached gingiva in areas without keratinized tissue by MARF flap. Their results showed a significant increase in the width of keratinized tissue and attached gingiva and also showed no increase in probe depth and no gingival recession was observed (Carnio et al., 2020). Their results were also in line with our results. However, the increase in thickness and width of the attached gingiva was significantly greater in the PRF group than in the MARF group.

The MARF and PRF groups resulted in a significant increase in keratinized tissue. MARF is an effective and efficient method to increase keratinized tissue and the attached gingival width. The main limitation of this technique is the presence of at least 0.5 mm of attached gingiva before surgery so that the wound is surrounded by keratinized tissue. The presence of bone dehiscence is another contraindication (Swarna et al., 2019). In the present study, it was shown that the thickness of tissue after surgery in the PRF method is significantly higher than in the MARF group. The possible reason is due to the nature of the PRF membrane, which is a three‐dimensional fibrin network that allows endothelial cells to proliferate within. Growth factors such as vascular endothelial growth factor and insulin‐like growth factor also accelerate angiogenesis (Choukroun et al., 2006b). The result leads to more vascular and thicker granulation tissue. The results show that the thickness increased by an average of 0.29 mm in the PRF group compared to the baseline, while this amount was reported to be 0.11 mm in the MARF group. Due to the significant increase in tissue thickness, the presence of growth factors, and promotion of wound healing, this method can also be used in cases of bone dehiscence.

Alaood et al. (2020) showed that the use of PRF in the treatment of gingival recession is not only useful but also has a greater effect on thickness compared to the control group, which used connective tissue graft with a coronally advanced flap.

Free gingival grafting is the most common method for increasing keratinized tissue and attached gingiva (Miller, 1993). The main disadvantages of FGG over MARF are the use of palatal tissue as a donor, the possibility of postoperative bleeding, color disparity between the grafted site and adjacent tissues, and increased morbidity (Carnio et al., 2015). In the study by Carnio et al. (2020), the MARF technique was performed in areas without the presence of keratinized tissue in the mid‐buccal site of the tooth and the results showed that the apicocoronal dimensions of keratinized and attached tissue increased significantly. There was no increase in pocket depth and no marginal gingival recession was reported. These results were also stable over the long term (Carnio et al., 2020). According to the findings of this study, after 2 months of follow‐up, keratinized tissue increased by 1.7 mm in the MARF treatment group and by 2.3 mm in the PRF treatment group. This increase was significant in either group, but the PRF group was higher. In both groups, the probing pocket depth and margin of the gingiva position were not changed.

Temmerman et al. (2018) in a randomized controlled trial examined the increase in keratinized width around the implant by using PRF. They tried to increase keratinized tissue, on the one had, using FGG, and, on the other hand, using an apically repositioned flap with PRF. The obtained keratinized tissue was significantly higher in the FGG group than in the PRF group. Also, shrinkage was higher in the PRF group than in the FGG group. Postoperative pain and duration of surgery were significantly higher in the FGG group (Temmerman et al., 2018). In the present study, the keratinized tissue obtained in terms of width and thickness in the PRF group had a significant increase compared to the MARF group.

Postoperative pain was also slightly reported in both groups, with no significant difference. Inflammation peak and maximum postoperative pain occur within the first 24 h following surgery. Since the PRF membrane acts as a biological dressing, it was expected to be effective in reducing postoperative pain. Several studies demonstrate that PRF decreases postoperative pain (de Almeida Barros Mourão et al., 2020; Fujioka‐Kobayashi et al., 2021; Bennardo et al., 2021; Gülşen & Şentürk, 2017). The possible reasons for this lack of reduction may be the following: the presence of more periosteal sutures for fixation of the PRF membranes in the PRF group leads to more inflammation. Using adhesive dressing can reduce the number of sutures, so the VAS score may be reduced. Rinsing the mouth with chlorhexidine has a negative impact on PRF cells (Csönge et al., 2021). Isolating the PRF membrane with adhesive dressing from oral liquids or using less aggressive mouthwash may lead to better results.

Another possible reason is using silica‐coated blood collection tubes. Silica in the tube causes negative effects on periosteal cells (Masuki et al., 2020), clot formation (R. J. Miron et al., 2020), and regeneration (Tsujino et al., 2019). To overcome the negative impact of silica, chemical‐free glass tubes are recommended (R. J. Miron et al., 2021).

In the PRF group, wound shrinkage was significantly lower than in the MARF group. These values were reported to be 30.9% for the PRF group and 51.1% for the MARF group. PRF group shrinkage values are comparable to those of Temmerman et al. (2018).

According to the shrinkage results, for obtaining at least 2 mm of new keratinized tissue, we suggest a 4 mm apically repositioned flap in MARF and a 3 mm repositioned flap in the MARF + PRF method.

Recent advances in the field of platelet derivatives lead to the use of low‐speed centrifugation concept (LSCC) and horizontal centrifugation. LSCC results in an increased growth factor concentration. Therefore, enhancing the growth factor concentrations improves the potential of tissue regeneration and wound healing capacity of the fluid PRF‐based matrices (Choukroun & Ghanaati, 2018). Horizontal centrifugation was proposed as a means to better separate blood cell layers for the production of PRF, and higher concentration of platelets and leukocytes (R. J. Miron et al., 2019b).

Due to recent improvements in platelet derivatives, further clinical studies are necessary to evaluate LSCC and horizontal centrifugation to enhance clinical results.

## 5 LIMITATION

This study was limited to the mandibular premolar area due to the split‐mouth design. Also, a small sample size may be subject to selection bias. Wound shrinkage will continue for months and the complete shrinkage assessed needs long‐term follow‐ups. Pain assessment one day after surgery may lead to bias.

To substantiate our findings, further and multicentered studies are needed.

## 6 CONCLUSION

Using PRF with the MARF method significantly increased the width and thickness of the gingiva and reduced shrinkage compared to MARF without PRF. Postoperative pain scores and VD changes were similar in the test and control groups. Increasing the GT may allow PRF to be used in the presence of bone dehiscence.

## AUTHOR CONTRIBUTIONS

**Torkzaban Parviz**: Conceptualization (equal); writing – original draft (supporting); methodology (equal). **Nazli Rabienejad**: Conceptualization (supporting); writing – original draft (supporting); methodology (equal). **Zahra Cheraghi**: Formal analysis (lead) **Kamran Lahoorpoor**: Conceptualization (equal); writing – original draft (lead); writing – review and editing (lead); methodology (equal).

## CONFLICT OF INTEREST

The authors declare no conflict of interest.

## ETHICS STATEMENT

The study protocol was registered on the IRCT registry, with Registration reference: IRCT20211124053173N1.

## Data Availability

The applicant has unlimited access to the data after sending the request via email to the corresponding author.
